# Applying postcolonial theory in academic medicine

**DOI:** 10.1177/01410768221084081

**Published:** 2022-03-09

**Authors:** Ahmed Rashid, Cynthia Whitehead

**Affiliations:** 1 UCL Medical School, Royal Free Hospital, London, NW3 2PR, UK; 2 Department of Family and Community Medicine, Temerty Faculty of Medicine, University of Toronto, ON, Canada; 3 Wilson Centre, University Health Network, University of Toronto, 200 Elizabeth Street, 1ES-565 Toronto, Ontario, Canada M5G 2C4

Seventeenth-century physician–philosopher John Locke once said ‘reading furnishes the mind only with materials of knowledge; it is thinking that makes what we read ours’.^1^ There is growing recognition within academic medicine that we must think about the texts we read in ways that acknowledge enduring racial and global inequities. Postcolonial theory offers a powerful lens to do this.

Bleakley et al. introduced postcolonial theory to the field of medical education in 2008,^2^ although notions of egalitarianism and social justice go back much further in the field.^3^ Other scholars have begun to follow suit, drawing on postcolonialism and other related concepts including decoloniality.^4^ Here we focus on postcolonialism, an approach frequently applied in literature and social sciences.^5^ Postcolonialism draws attention to how language is used to legitimise actions and perspectives that allow the historical, political, economic, cultural and social impacts of European colonial rule to remain dominant in the world today.

Edward Said’s work has been foundational to the development of postcolonialism as an academic discipline. In his landmark book, *Orientalism*, he painted a vivid picture of how many literary ‘greats’ of the English and French languages perpetuated racial stereotypes of Eastern cultures and peoples in their novels, ultimately serving to consolidate colonial power.^6^ In his later book *Culture and Imperialism*, Said demonstrates that this cultural dominance is regularly met with resistance.^7^ Indeed, he argues that European culture needs to be read in relation to the works that colonised people themselves produced in reaction to cultural domination. He describes this as ‘contrapuntal’ reading, borrowing the term from classical musical scholars’ description of listening for ‘counterpoint’. In essence, it refers to reading a text with a simultaneous awareness both of the metropolitan history that is narrated, and of those of other histories against which, and together with which, the dominant history runs. In other words, instead of reading a text in a superficial and idealised way, one should recognise both the colonising and colonised perspectives across an ‘imperial divide’.^7^

How might we engage in such contrapuntal reading of the variety of texts we encounter in medicine and medical education? For example, how might this help readers approach a student application statement, a trainee reflective essay, or a programme or institution accreditation report? Are we able to identify embedded assumptions in these texts that serve to perpetuate Western dominance? In our globalised world, most faculties and student bodies are made up of individuals from a variety of different racial and cultural backgrounds, and the use of a deliberate lens to identify statements of resistance from marginalised voices could help empower and embolden those seeking to challenge oppression in its many forms. However, contrapuntal reading has perhaps even more relevance when broadening our perspectives from individual departments and institutions and considers the ways in which medical education is becoming more interconnected and integrated in a global way. How, for example, might one contrapuntally read the experiences of medical educators in Eastern parts of the world who are adopting Western approaches, or working with Western ‘donor’ or ‘supplier’ institutions? Or how might one read global consensus statements or standards that are designed to be used in all parts of the world?

In their article drawing on postcolonial thinking, Bleakley et al. lament the universalisation of medical education, likening it to the cultural phenomenon of ‘McDonaldisation’.^2^ Such ‘universalisation’ and ‘globalisation’ often implicitly foreground Western perspectives. The encouragement of national accreditation agencies to engage in Western style accreditation practices through the World Federation for Medical Education recognition programme is a prime example.^8^ A contrapuntal reading, though, might actively amplify voices that see these movements as destructive due to ‘brain drain’ and workforce shortages, or else those who see their local cultures and values being marginalised.^9^ As Said recognised, such contrapuntal reading necessitates a ‘stubborn confrontation’ with normative practices and can, therefore, be less than comfortable.^6^

As Locke highlighted, each reading of a text involves some interaction with it, and importantly, some thinking. What are we provoked to think, and therefore do, as a result of this reading? Contrapuntal reading forces us to ensure that we do not take words at face value, but rather scratch the surface and identify the unintended consequences of particular statements and ideas. More specifically, it forces us to recognise the inescapable realities of colonialism and its enduring effects on nations, institutions, cultures and individuals. What is critically important, though, is that we do not stop at reading and thinking. Having engaged contrapuntally with texts and recognised the voices of the marginalised, we must channel and strengthen these voices and work to find policy solutions that recognise power imbalances and seek to ameliorate them.

## Declarations

### Guarantor

AR.

## References

[1] Locke J . Reading furnishes the mind only with materials of knowledge. The Saturday Magazine 1836; 8: 186–186.

[2] Bleakley A Brice J Bligh J . Thinking the post-colonial in medical education. Med Educ 2008; 42: 266–270. 18275413 10.1111/j.1365-2923.2007.02991.x

[3] Sanazaro PJ . An agenda for research in medical education. JAMA 1966; 197: 979–984. 5953214

[4] Naidu T. Southern exposure: levelling the Northern tilt in global medical and medical humanities education. *Advances in Health Sciences Education* 2021; 26: 739–752. 10.1007/s10459-020-09976-9 32500281

[5] Young RJ . Postcolonialism: An Historical Introduction, Hoboken, NJ: John Wiley & Sons, 2016.

[6] Said EW. *Orientalism*. New York: Vintage, 1979.

[7] Said EW. *Culture and Imperialism*. New York: Vintage, 1993.

[8] World Federation for Medical Education. WFME recognition programme. See http://wfme.org/accreditation/recognition-programme (last checked 18 February 2022).

[9] Rashid MA. *Global approaches to medical school regulation: a critical discourse analysis*. Doctoral dissertation, University College London, UK.

